# Supplementary material for: Clostridium butyricum RH2 ameliorates diarrhea in juvenile mice under continuous antibiotic exposure by modulating gut microbiota and metabolome
Source: Microbiol Spectr. 2026 Jan 7;14(2):e01976-25. doi: 10.1128/spectrum.01976-25 (PMC12889019; doi:10.1128/spectrum.01976-25)
Supplement: Supplemental material — Table S1; Fig. S1 to S3. [file spectrum.01976-25-s0001.docx]

Supplementary data

Supplementary Table 1. Primers used for Quantitative-PCR analysis

| Target mRNA | Direction Sequence  (Forward) | Direction Sequence  (Reverse) |
| --- | --- | --- |
| Glyceraldehyde-3-phosphate dehydrogenase (Gapdh) | AGGTCGGTGTGAACGGATTTG | GGGGTCGTTGATGGCAACA |
| Claudin 1(Cldn1) | ATGCCTTCAACTGTTCTGTATCTC | AATCCAGGTCTACCAATGTCAATG |
| Claudin 5 (Cldn5) | GCTCTCAGAGTCCGTTGACC | CTGCCCTTTCAGGTTAGCAG |
| Occludin (Ocln) | CTGCTGCTGATGAATATAATAG | CCTCTTGATGTGCGATAA |
| Tight junction protein 1  (Tjp1/ZO-1) | CATAGTTCAACACAGCCTCCAG | CCATCCTCATCTTCATCTTCTTCC |
| Cadherin 1 (Cdh1) | TTGTTCGGCTATGTGTCT | TGTGTACCTAAGAATCTGAGA |
| Mucin 2 (Muc-2) | GATGGCACCTACCTCGTTGT | GTCCTGGCACTTGTTGGAAT |
| Interleukin 6 (Il6) | CTGCAAGAGACTTCCATCCAG | AGTGGTATAGACAGGTCTGTTGG |
| Tumor necrosis factor α（Tnfα） | CAGGCGGTGCCTATGTCTC | CGATCACCCCGAAGTTCAGTAG |
| Interleukin 10（Il10） | CTTACTGACTGGCATGAGGATCA | GCAGCTCTAGGAGCATGTGG |
| Interleukin 1 beta (Il1β) | GAAATGCCACCTTTTGACAGTG | TGGATGCTCTCATCAGGACAG |
| Interleukin 4 (Il4) | GGTCTCAACCCCCAGCTAGT | GCCGATGATCTCTCTCAAGTGAT |

Supplemental Figure 1


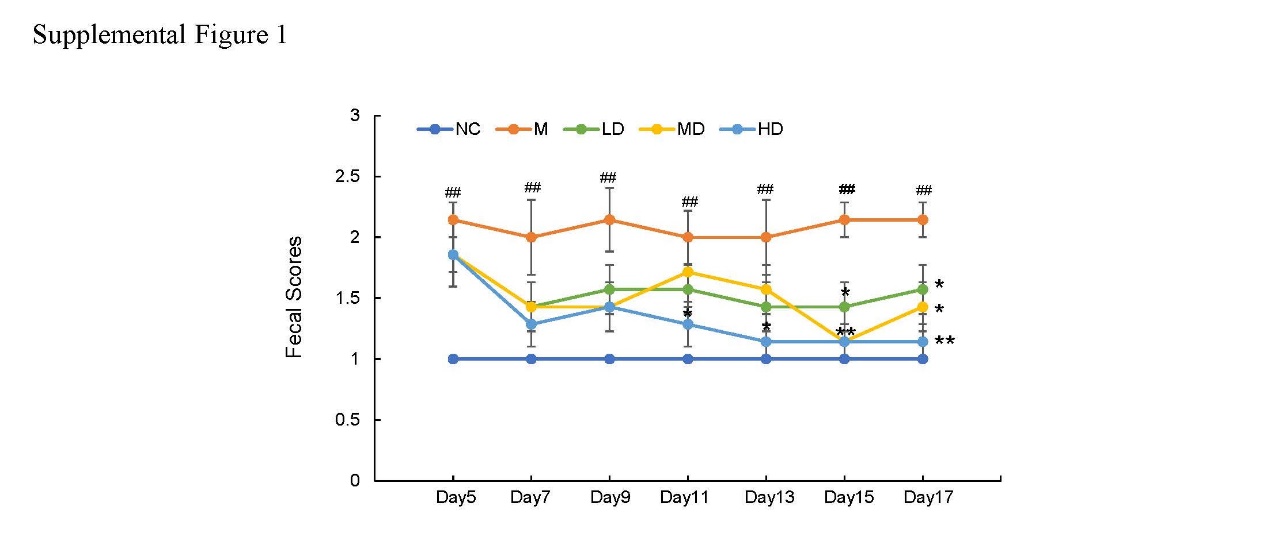


Changes of diarrhea status score in mice. Data were presented as the means ± SEM, * *p* < 0.05, ** *p* < 0 .01 vs. M; ## *p* < 0.01 vs. NC.

Supplemental Figure 2


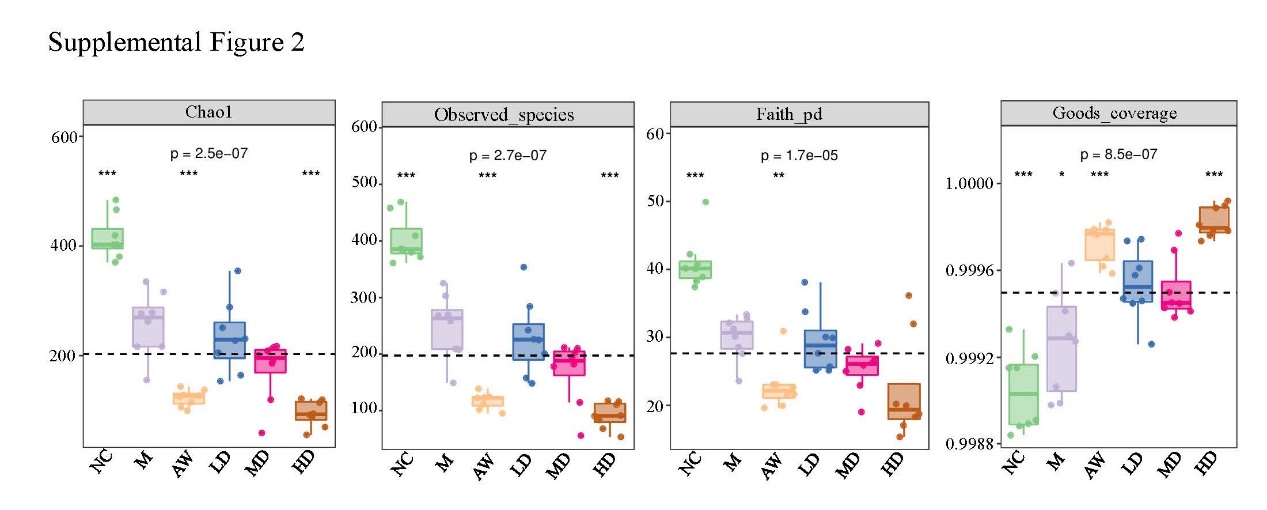


Alpha diversity of the gut microbiota.

Supplemental Figure 3


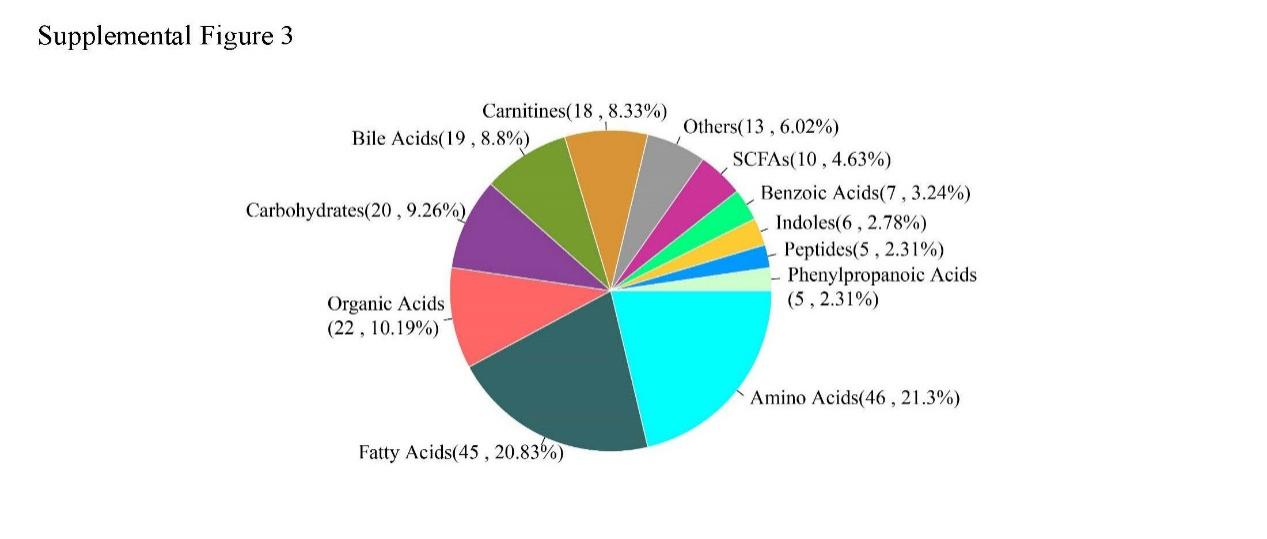


The summary of metabolite counts in each metabolite class is shown in a pile chart.
